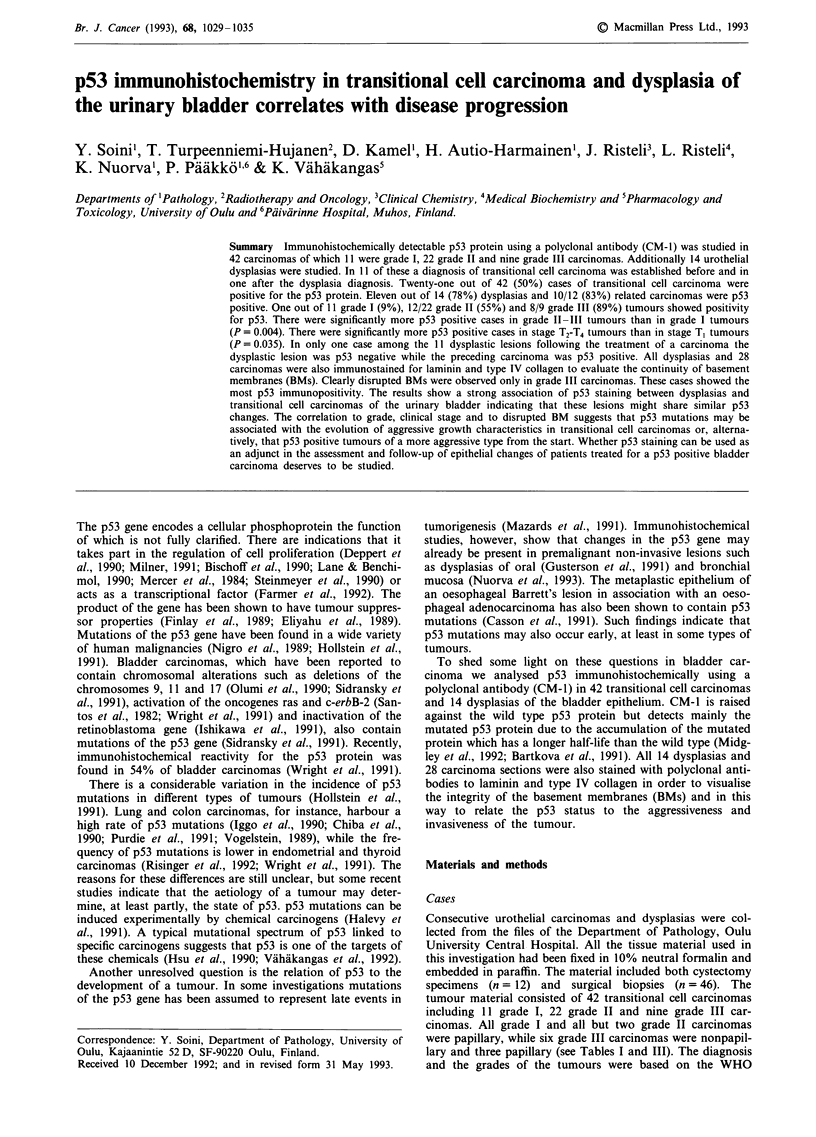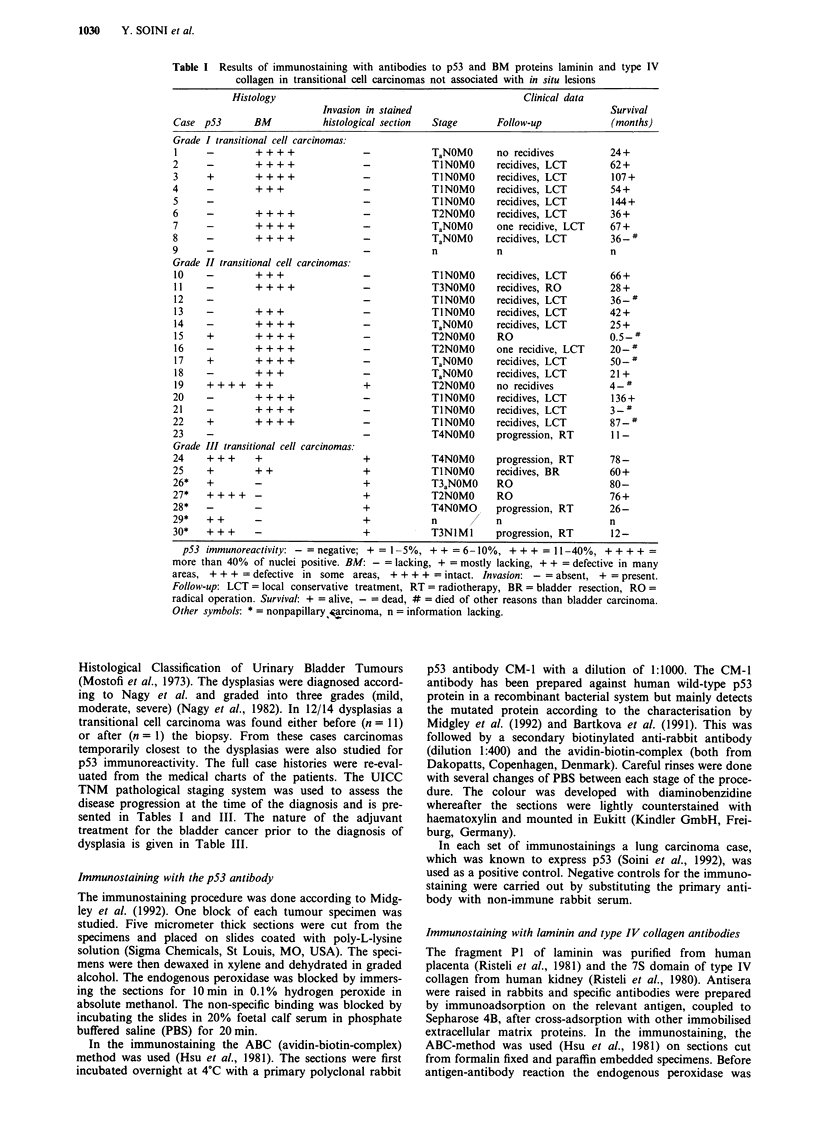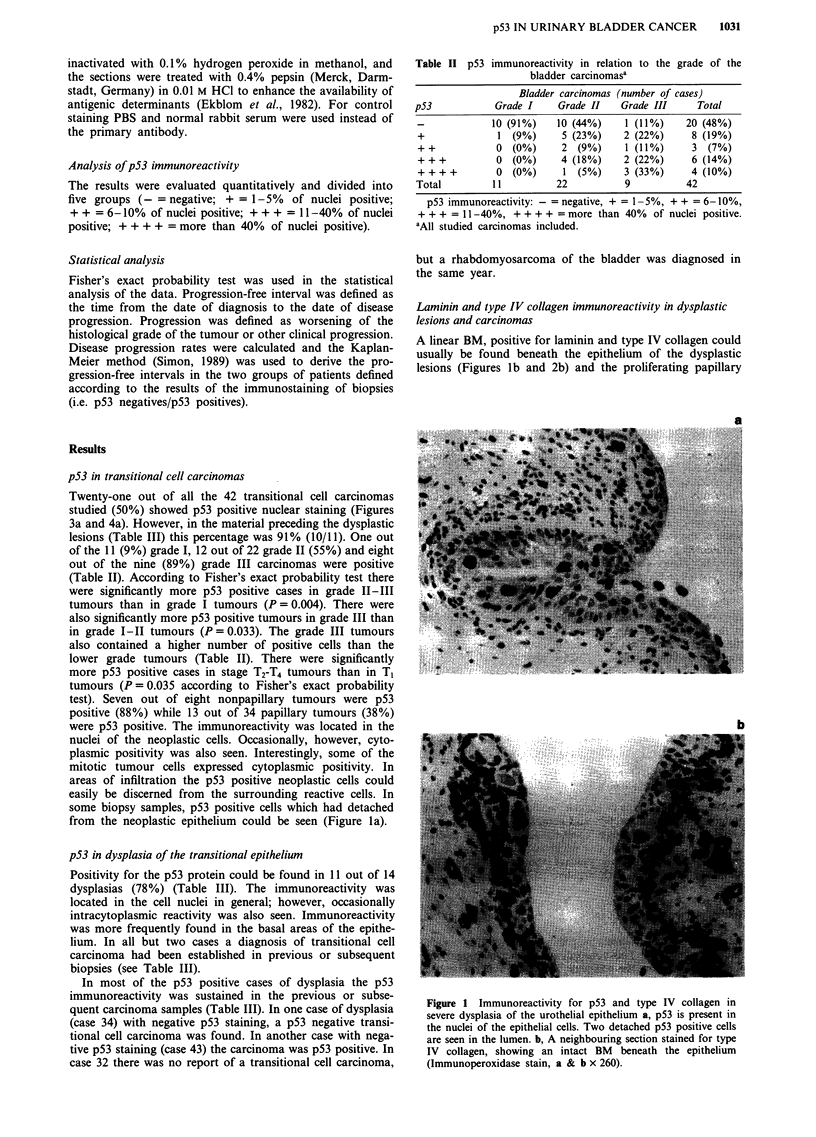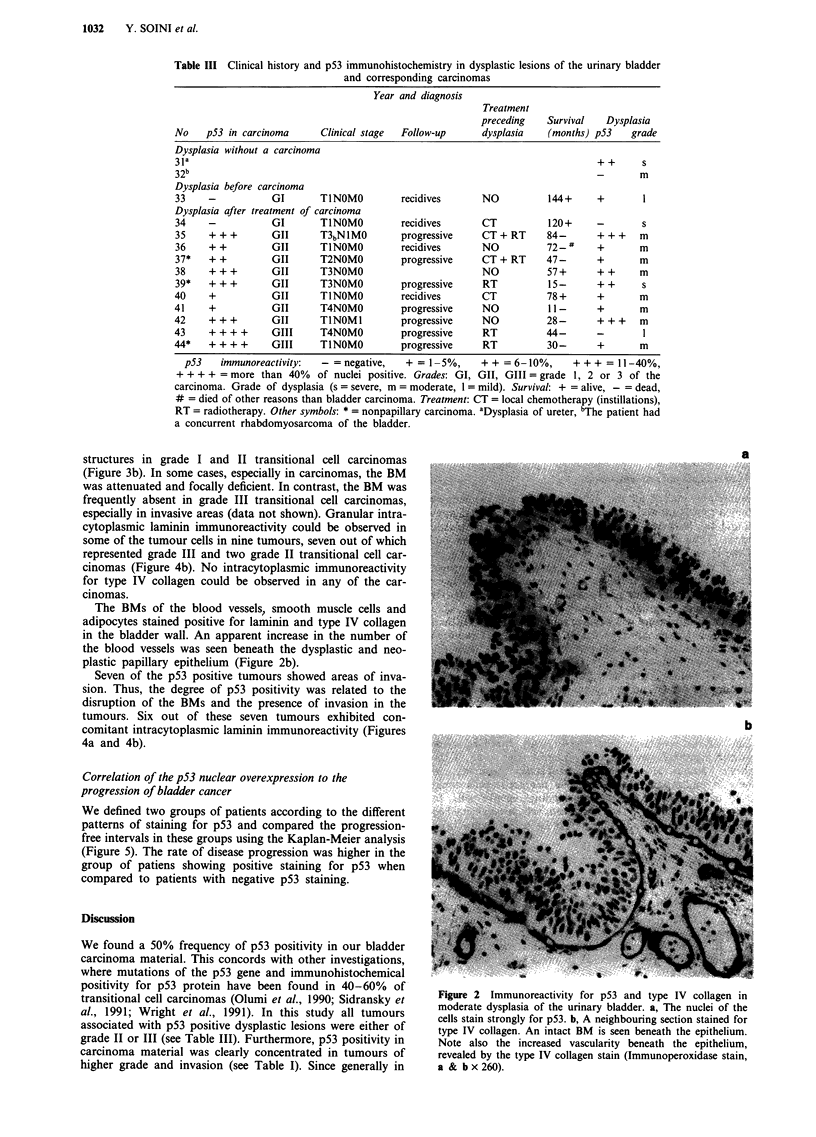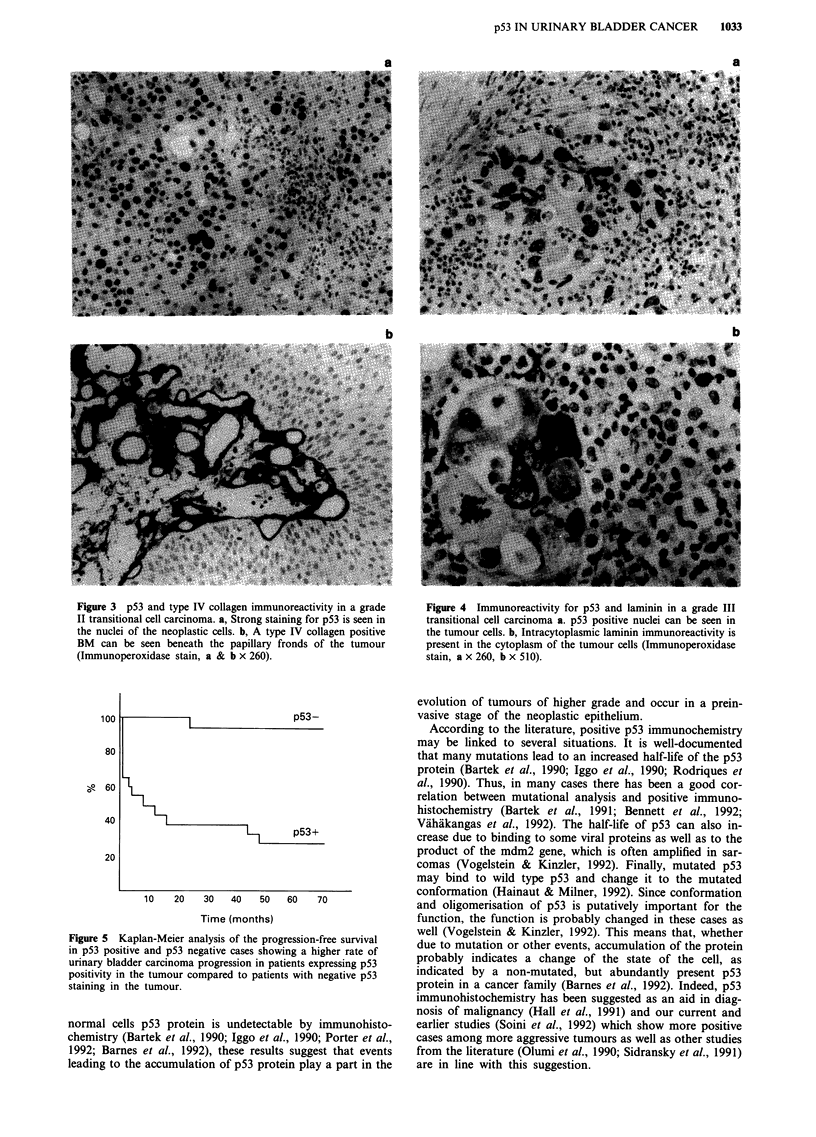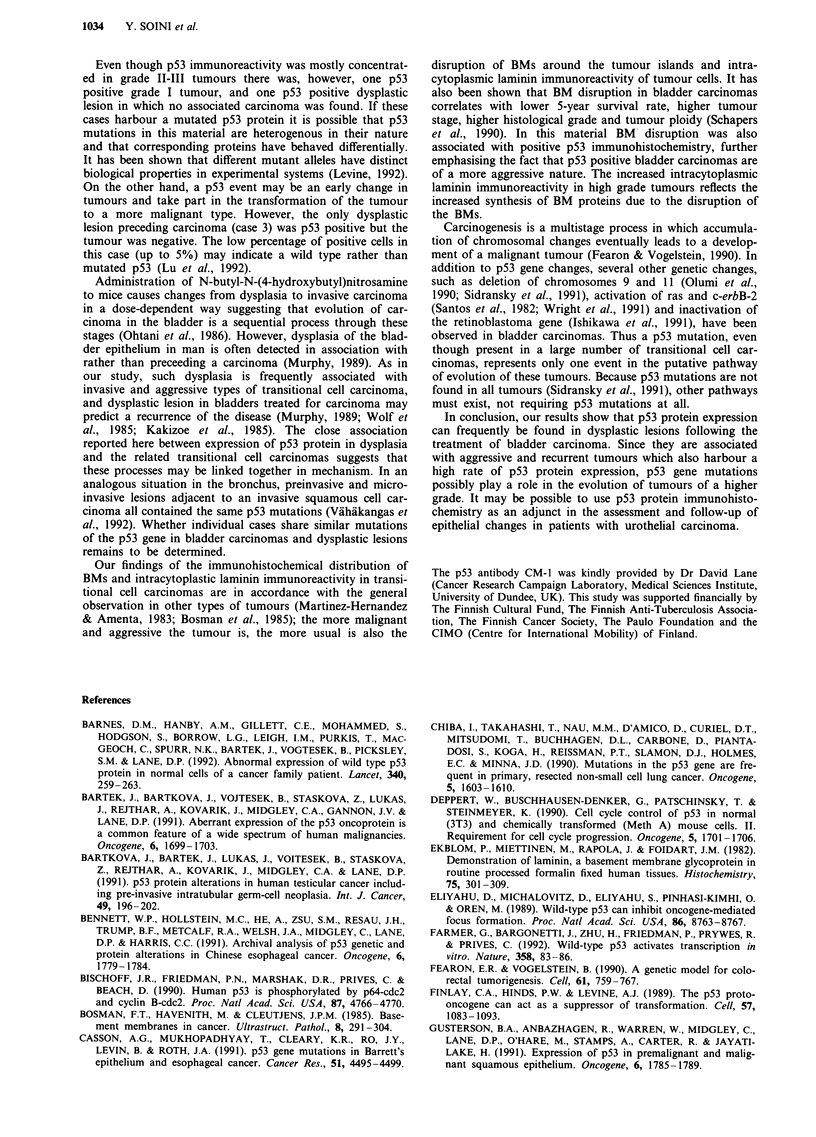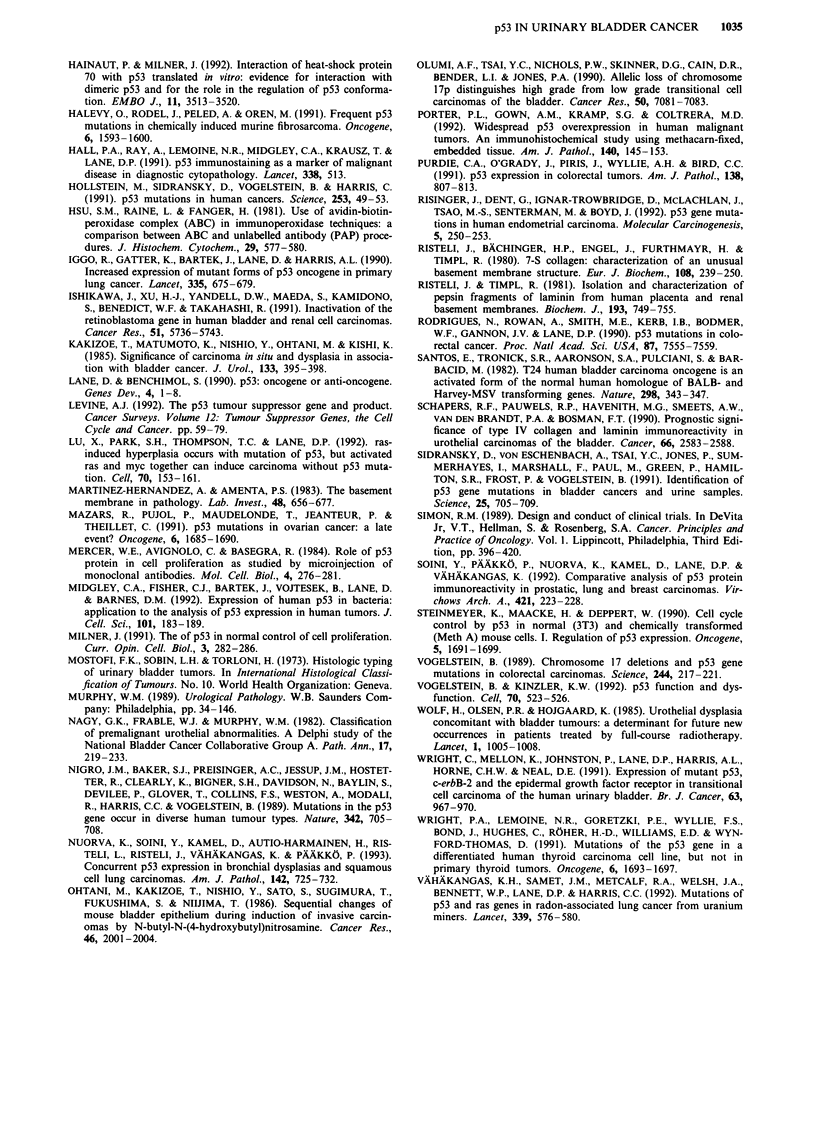# p53 immunohistochemistry in transitional cell carcinoma and dysplasia of the urinary bladder correlates with disease progression.

**DOI:** 10.1038/bjc.1993.475

**Published:** 1993-11

**Authors:** Y. Soini, T. Turpeenniemi-Hujanen, D. Kamel, H. Autio-Harmainen, J. Risteli, L. Risteli, K. Nuorva, P. Pääkkö, K. Vähäkangas

**Affiliations:** Department of Pathology, University of Oulu, Finland.

## Abstract

**Images:**


Br. J. Cancer (1993), 68, 1029–1035 © Macmillan Press Ltd., 1993

### p53 immunohistochemistry in transitional cell carcinoma and dysplasia of the urinary bladder correlates with disease progression

Y. Soini[1], T. Turpeenniemi-Hujanen[2], D. Kamel[1], H. Autio-Harmainen[1], J. Risteli[3], L. Risteli[4], K. Nuorva[1], P. Pääkkö[1,6] & K. Vähäkangas[5]

*Departments of [1]Pathology, [2]Radiotherapy and Oncology, [3]Clinical Chemistry, [4]Medical Biochemistry and [5]Pharmacology and Toxicology, University of Oulu and [6]Päivärinne Hospital, Muhos, Finland.*

**Summary** Immunohistochemically detectable p53 protein using a polyclonal antibody (CM-1) was studied in 42 carcinomas of which 11 were grade I, 22 grade II and nine grade III carcinomas. Additionally 14 urothelial dysplasias were studied. In 11 of these a diagnosis of transitional cell carcinoma was established before and in one after the dysplasia diagnosis. Twenty-one out of 42 (50%) cases of transitional cell carcinoma were positive for the p53 protein. Eleven out of 14 (78%) dysplasias and 10/12 (83%) related carcinomas were p53 positive. One out of 11 grade I (9%), 12/22 grade II (55%) and 8/9 grade III (89%) tumours showed positivity for p53. There were significantly more p53 positive cases in grade II–III tumours than in grade I tumours ($P = 0.004$). There were significantly more p53 positive cases in stage $T_2$-$T_4$ tumours than in stage $T_1$ tumours ($P = 0.035$). In only one case among the 11 dysplastic lesions following the treatment of a carcinoma the dysplastic lesion was p53 negative while the preceding carcinoma was p53 positive. All dysplasias and 28 carcinomas were also immunostained for laminin and type IV collagen to evaluate the continuity of basement membranes (BMs). Clearly disrupted BMs were observed only in grade III carcinomas. These cases showed the most p53 immunopositivity. The results show a strong association of p53 staining between dysplasias and transitional cell carcinomas of the urinary bladder indicating that these lesions might share similar p53 changes. The correlation to grade, clinical stage and to disrupted BM suggests that p53 mutations may be associated with the evolution of aggressive growth characteristics in transitional cell carcinomas or, alternatively, that p53 positive tumours of a more aggressive type from the start. Whether p53 staining can be used as an adjunct in the assessment and follow-up of epithelial changes of patients treated for a p53 positive bladder carcinoma deserves to be studied.

The p53 gene encodes a cellular phosphoprotein the function of which is not fully clarified. There are indications that it takes part in the regulation of cell proliferation (Deppert *et al.*, 1990; Milner, 1991; Bischoff *et al.*, 1990; Lane & Benchimol, 1990; Mercer *et al.*, 1984; Steinmeyer *et al.*, 1990) or acts as a transcriptional factor (Farmer *et al.*, 1992). The product of the gene has been shown to have tumour suppressor properties (Finlay *et al.*, 1989; Eliyahu *et al.*, 1989). Mutations of the p53 gene have been found in a wide variety of human malignancies (Nigro *et al.*, 1989; Hollstein *et al.*, 1991). Bladder carcinomas, which have been reported to contain chromosomal alterations such as deletions of the chromosomes 9, 11 and 17 (Olumi *et al.*, 1990; Sidransky *et al.*, 1991), activation of the oncogenes ras and c-*erb*B-2 (Santos *et al.*, 1982; Wright *et al.*, 1991) and inactivation of the retinoblastoma gene (Ishikawa *et al.*, 1991), also contain mutations of the p53 gene (Sidransky *et al.*, 1991). Recently, immunohistochemical reactivity for the p53 protein was found in 54% of bladder carcinomas (Wright *et al.*, 1991).

There is a considerable variation in the incidence of p53 mutations in different types of tumours (Hollstein *et al.*, 1991). Lung and colon carcinomas, for instance, harbour a high rate of p53 mutations (Iggo *et al.*, 1990; Chiba *et al.*, 1990; Purdie *et al.*, 1991; Vogelstein, 1989), while the frequency of p53 mutations is lower in endometrial and thyroid carcinomas (Risinger *et al.*, 1992; Wright *et al.*, 1991). The reasons for these differences are still unclear, but some recent studies indicate that the aetiology of a tumour may determine, at least partly, the state of p53. p53 mutations can be induced experimentally by chemical carcinogens (Halevy *et al.*, 1991). A typical mutational spectrum of p53 linked to specific carcinogens suggests that p53 is one of the targets of these chemicals (Hsu *et al.*, 1990; Vähäkangas *et al.*, 1992).

Another unresolved question is the relation of p53 to the development of a tumour. In some investigations mutations of the p53 gene has been assumed to represent late events in tumorigenesis (Mazards *et al.*, 1991). Immunohistochemical studies, however, show that changes in the p53 gene may already be present in premalignant non-invasive lesions such as dysplasias of oral (Gusterson *et al.*, 1991) and bronchial mucosa (Nuorva *et al.*, 1993). The metaplastic epithelium of an oesophageal Barrett's lesion in association with an oesophageal adenocarcinoma has also been shown to contain p53 mutations (Casson *et al.*, 1991). Such findings indicate that p53 mutations may also occur early, at least in some types of tumours.

To shed some light on these questions in bladder carcinoma we analysed p53 immunohistochemically using a polyclonal antibody (CM-1) in 42 transitional cell carcinomas and 14 dysplasias of the bladder epithelium. CM-1 is raised against the wild type p53 protein but detects mainly the mutated p53 protein due to the accumulation of the mutated protein which has a longer half-life than the wild type (Midgley *et al.*, 1992; Bartkova *et al.*, 1991). All 14 dysplasias and 28 carcinoma sections were also stained with polyclonal antibodies to laminin and type IV collagen in order to visualise the integrity of the basement membranes (BMs) and in this way to relate the p53 status to the aggressiveness and invasiveness of the tumour.

### Materials and methods

### *Cases*

Consecutive urothelial carcinomas and dysplasias were collected from the files of the Department of Pathology, Oulu University Central Hospital. All the tissue material used in this investigation had been fixed in 10% neutral formalin and embedded in paraffin. The material included both cystectomy specimens ($n = 12$) and surgical biopsies ($n = 46$). The tumour material consisted of 42 transitional cell carcinomas including 11 grade I, 22 grade II and nine grade III carcinomas. All grade I and all but two grade II carcinomas were papillary, while six grade III carcinomas were nonpapillary and three papillary (see Tables I and III). The diagnosis and the grades of the tumours were based on the WHO

Correspondence: Y. Soini, Department of Pathology, University of Oulu, Kajaanintie 52 D, SF-90220 Oulu, Finland.
Received 10 December 1992; and in revised form 31 May 1993.

**Table I** Results of immunostaining with antibodies to p53 and BM proteins laminin and type IV collagen in transitional cell carcinomas not associated with *in situ* lesions

| *Histology* | | | | *Clinical data* | | |
|---|---|---|---|---|---|---|
| *Case* | *p53* | *BM* | *Invasion in stained histological section* | *Stage* | *Follow-up* | *Survival (months)* |
| *Grade I transitional cell carcinomas:* | | | | | | |
| 1 | – | + + + + | – | $T_a$N0M0 | no recidives | 24+ |
| 2 | – | + + + + | – | T1N0M0 | recidives, LCT | 62+ |
| 3 | + | + + + + | – | T1N0M0 | recidives, LCT | 107+ |
| 4 | – | + + + | – | T1N0M0 | recidives, LCT | 54+ |
| 5 | – | | – | T1N0M0 | recidives, LCT | 144+ |
| 6 | – | + + + + | – | T2N0M0 | recidives, LCT | 36+ |
| 7 | – | + + + + | – | $T_a$N0M0 | one recidive, LCT | 67+ |
| 8 | – | + + + + | – | $T_a$N0M0 | recidives, LCT | 36–# |
| 9 | – | | – | n | n | n |
| *Grade II transitional cell carcinomas:* | | | | | | |
| 10 | – | + + + | – | T1N0M0 | recidives, LCT | 66+ |
| 11 | – | + + + + | – | T3N0M0 | recidives, RO | 28+ |
| 12 | – | | – | T1N0M0 | recidives, LCT | 36–# |
| 13 | – | + + + | – | T1N0M0 | recidives, LCT | 42+ |
| 14 | – | + + + + | – | $T_a$N0M0 | recidives, LCT | 25+ |
| 15 | + | + + + + | – | T2N0M0 | RO | 0.5–# |
| 16 | – | + + + + | – | T2N0M0 | one recidive, LCT | 20–# |
| 17 | + | + + + + | – | $T_a$N0M0 | recidives, LCT | 50–# |
| 18 | – | + + + | – | $T_a$N0M0 | recidives, LCT | 21+ |
| 19 | + + + + | + + | + | T2N0M0 | no recidives | 4–# |
| 20 | – | + + + + | – | T1N0M0 | recidives, LCT | 136+ |
| 21 | – | + + + + | – | T1N0M0 | recidives, LCT | 3–# |
| 22 | + | + + + + | – | T1N0M0 | recidives, LCT | 87–# |
| 23 | – | | – | T4N0M0 | progression, RT | 11– |
| *Grade III transitional cell carcinomas:* | | | | | | |
| 24 | + + + | + | + | T4N0M0 | progression, RT | 78– |
| 25 | + | + + | + | T1N0M0 | recidives, BR | 60+ |
| 26* | + | – | + | $T3_a$N0M0 | RO | 80– |
| 27* | + + + + | – | + | T2N0M0 | RO | 76+ |
| 28* | – | – | + | T4N0M0 | progression, RT | 26– |
| 29* | + + | – | + | n | n | n |
| 30* | + + + | – | + | T3N1M1 | progression, RT | 12– |

*p53 immunoreactivity*: – = negative; + = 1–5%, + + = 6–10%, + + + = 11–40%, + + + + = more than 40% of nuclei positive. *BM*: – = lacking, + = mostly lacking, + + = defective in many areas, + + + = defective in some areas, + + + + = intact. *Invasion*: – = absent, + = present. *Follow-up*: LCT = local conservative treatment, RT = radiotherapy, BR = bladder resection, RO = radical operation. *Survival*: + = alive, – = dead, # = died of other reasons than bladder carcinoma. *Other symbols*: * = nonpapillary carcinoma, n = information lacking.

Histological Classification of Urinary Bladder Tumours (Mostofi *et al.*, 1973). The dysplasias were diagnosed according to Nagy *et al.* and graded into three grades (mild, moderate, severe) (Nagy *et al.*, 1982). In 12/14 dysplasias a transitional cell carcinoma was found either before ($n = 11$) or after ($n = 1$) the biopsy. From these cases carcinomas temporarily closest to the dysplasias were also studied for p53 immunoreactivity. The full case histories were re-evaluated from the medical charts of the patients. The UICC TNM pathological staging system was used to assess the disease progression at the time of the diagnosis and is presented in Tables I and III. The nature of the adjuvant treatment for the bladder cancer prior to the diagnosis of dysplasia is given in Table III.

### *Immunostaining with the p53 antibody*

The immunostaining procedure was done according to Midgley *et al.* (1992). One block of each tumour specimen was studied. Five micrometer thick sections were cut from the specimens and placed on slides coated with poly-L-lysine solution (Sigma Chemicals, St Louis, MO, USA). The specimens were then dewaxed in xylene and dehydrated in graded alcohol. The endogenous peroxidase was blocked by immersing the sections for 10 min in 0.1% hydrogen peroxide in absolute methanol. The non-specific binding was blocked by incubating the slides in 20% foetal calf serum in phosphate buffered saline (PBS) for 20 min.

In the immunostaining the ABC (avidin-biotin-complex) method was used (Hsu *et al.*, 1981). The sections were first incubated overnight at 4°C with a primary polyclonal rabbit p53 antibody CM-1 with a dilution of 1:1000. The CM-1 antibody has been prepared against human wild-type p53 protein in a recombinant bacterial system but mainly detects the mutated protein according to the characterisation by Midgley *et al.* (1992) and Bartkova *et al.* (1991). This was followed by a secondary biotinylated anti-rabbit antibody (dilution 1:400) and the avidin-biotin-complex (both from Dakopatts, Copenhagen, Denmark). Careful rinses were done with several changes of PBS between each stage of the procedure. The colour was developed with diaminobenzidine whereafter the sections were lightly counterstained with haematoxylin and mounted in Eukitt (Kindler GmbH, Freiburg, Germany).

In each set of immunostainings a lung carcinoma case, which was known to express p53 (Soini *et al.*, 1992), was used as a positive control. Negative controls for the immunostaining were carried out by substituting the primary antibody with non-immune rabbit serum.

### *Immunostaining with laminin and type IV collagen antibodies*

The fragment P1 of laminin was purified from human placenta (Risteli *et al.*, 1981) and the 7S domain of type IV collagen from human kidney (Risteli *et al.*, 1980). Antisera were raised in rabbits and specific antibodies were prepared by immunoadsorption on the relevant antigen, coupled to Sepharose 4B, after cross-adsorption with other immobilised extracellular matrix proteins. In the immunostaining, the ABC-method was used (Hsu *et al.*, 1981) on sections cut from formalin fixed and paraffin embedded specimens. Before antigen-antibody reaction the endogenous peroxidase was inactivated with 0.1% hydrogen peroxide in methanol, and the sections were treated with 0.4% pepsin (Merck, Darmstadt, Germany) in 0.01 M HCl to enhance the availability of antigenic determinants (Ekblom *et al.*, 1982). For control staining PBS and normal rabbit serum were used instead of the primary antibody.

*Analysis of p53 immunoreactivity*

The results were evaluated quantitatively and divided into five groups (− = negative; + = 1–5% of nuclei positive; + + = 6–10% of nuclei positive; + + + = 11–40% of nuclei positive; + + + + = more than 40% of nuclei positive).

*Statistical analysis*

Fisher's exact probability test was used in the statistical analysis of the data. Progression-free interval was defined as the time from the date of diagnosis to the date of disease progression. Progression was defined as worsening of the histological grade of the tumour or other clinical progression. Disease progression rates were calculated and the Kaplan-Meier method (Simon, 1989) was used to derive the progression-free intervals in the two groups of patients defined according to the results of the immunostaining of biopsies (i.e. p53 negatives/p53 positives).

### Results

*p53 in transitional cell carcinomas*

Twenty-one out of all the 42 transitional cell carcinomas studied (50%) showed p53 positive nuclear staining (Figures 3a and 4a). However, in the material preceding the dysplastic lesions (Table III) this percentage was 91% (10/11). One out of the 11 (9%) grade I, 12 out of 22 grade II (55%) and eight out of the nine (89%) grade III carcinomas were positive (Table II). According to Fisher's exact probability test there were significantly more p53 positive cases in grade II–III tumours than in grade I tumours ($P = 0.004$). There were also significantly more p53 positive tumours in grade III than in grade I–II tumours ($P = 0.033$). The grade III tumours also contained a higher number of positive cells than the lower grade tumours (Table II). There were significantly more p53 positive cases in stage $T_2$-$T_4$ tumours than in $T_1$ tumours ($P = 0.035$ according to Fisher's exact probability test). Seven out of eight nonpapillary tumours were p53 positive (88%) while 13 out of 34 papillary tumours (38%) were p53 positive. The immunoreactivity was located in the nuclei of the neoplastic cells. Occasionally, however, cytoplasmic positivity was also seen. Interestingly, some of the mitotic tumour cells expressed cytoplasmic positivity. In areas of infiltration the p53 positive neoplastic cells could easily be discerned from the surrounding reactive cells. In some biopsy samples, p53 positive cells which had detached from the neoplastic epithelium could be seen (Figure 1a).

*p53 in dysplasia of the transitional epithelium*

Positivity for the p53 protein could be found in 11 out of 14 dysplasias (78%) (Table III). The immunoreactivity was located in the cell nuclei in general; however, occasionally intracytoplasmic reactivity was also seen. Immunoreactivity was more frequently found in the basal areas of the epithelium. In all but two cases a diagnosis of transitional cell carcinoma had been established in previous or subsequent biopsies (see Table III).

In most of the p53 positive cases of dysplasia the p53 immunoreactivity was sustained in the previous or subsequent carcinoma samples (Table III). In one case of dysplasia (case 34) with negative p53 staining, a p53 negative transitional cell carcinoma was found. In another case with negative p53 staining (case 43) the carcinoma was p53 positive. In case 32 there was no report of a transitional cell carcinoma, but a rhabdomyosarcoma of the bladder was diagnosed in the same year.

**Table II** p53 immunoreactivity in relation to the grade of the bladder carcinomas[a]

| | *Bladder carcinomas (number of cases)* | | | |
|---|---|---|---|---|
| *p53* | *Grade I* | *Grade II* | *Grade III* | *Total* |
| − | 10 (91%) | 10 (44%) | 1 (11%) | 20 (48%) |
| + | 1 (9%) | 5 (23%) | 2 (22%) | 8 (19%) |
| + + | 0 (0%) | 2 (9%) | 1 (11%) | 3 (7%) |
| + + + | 0 (0%) | 4 (18%) | 2 (22%) | 6 (14%) |
| + + + + | 0 (0%) | 1 (5%) | 3 (33%) | 4 (10%) |
| Total | 11 | 22 | 9 | 42 |

p53 immunoreactivity: − = negative, + = 1–5%, + + = 6–10%, + + + = 11–40%, + + + + = more than 40% of nuclei positive. [a]All studied carcinomas included.

*Laminin and type IV collagen immunoreactivity in dysplastic lesions and carcinomas*

A linear BM, positive for laminin and type IV collagen could usually be found beneath the epithelium of the dysplastic lesions (Figures 1b and 2b) and the proliferating papillary

**Figure 1** Immunoreactivity for p53 and type IV collagen in severe dysplasia of the urothelial epithelium **a**, p53 is present in the nuclei of the epithelial cells. Two detached p53 positive cells are seen in the lumen. **b**, A neighbouring section stained for type IV collagen, showing an intact BM beneath the epithelium (Immunoperoxidase stain, **a** & **b** × 260).

**Table III** Clinical history and p53 immunohistochemistry in dysplastic lesions of the urinary bladder and corresponding carcinomas

| | *Year and diagnosis* | | | | | | | |
|---|---|---|---|---|---|---|---|---|
| *No* | *p53 in carcinoma* | | *Clinical stage* | *Follow-up* | *Treatment preceding dysplasia* | *Survival (months)* | *Dysplasia p53* | *Dysplasia grade* |
| *Dysplasia without a carcinoma* | | | | | | | | |
| 31[a] | | | | | | | ++ | s |
| 32[b] | | | | | | | – | m |
| *Dysplasia before carcinoma* | | | | | | | | |
| 33 | – | GI | T1N0M0 | recidives | NO | 144+ | + | 1 |
| *Dysplasia after treatment of carcinoma* | | | | | | | | |
| 34 | – | GI | T1N0M0 | recidives | CT | 120+ | – | s |
| 35 | +++ | GII | $T3_bN1M0$ | progressive | CT + RT | 84– | +++ | m |
| 36 | ++ | GII | T1N0M0 | recidives | NO | 72–# | + | m |
| 37* | ++ | GII | T2N0M0 | progressive | CT + RT | 47– | + | m |
| 38 | +++ | GII | T3N0M0 | | NO | 57+ | ++ | m |
| 39* | +++ | GII | T3N0M0 | progressive | RT | 15– | ++ | s |
| 40 | + | GII | T1N0M0 | recidives | CT | 78+ | + | m |
| 41 | + | GII | T4N0M0 | progressive | NO | 11– | + | m |
| 42 | +++ | GII | T1N0M1 | progressive | NO | 28– | +++ | m |
| 43 | ++++ | GIII | T4N0M0 | progressive | RT | 44– | – | 1 |
| 44* | ++++ | GIII | T1N0M0 | progressive | RT | 30– | + | m |

*p53 immunoreactivity*: – = negative, + = 1–5%, ++ = 6–10%, +++ = 11–40%, ++++ = more than 40% of nuclei positive. *Grades*: GI, GII, GIII = grade 1, 2 or 3 of the carcinoma. Grade of dysplasia (s = severe, m = moderate, 1 = mild). *Survival*: + = alive, – = dead, # = died of other reasons than bladder carcinoma. *Treatment*: CT = local chemotherapy (instillations), RT = radiotherapy. *Other symbols*: * = nonpapillary carcinoma. [a]Dysplasia of ureter, [b]The patient had a concurrent rhabdomyosarcoma of the bladder.

structures in grade I and II transitional cell carcinomas (Figure 3b). In some cases, especially in carcinomas, the BM was attenuated and focally deficient. In contrast, the BM was frequently absent in grade III transitional cell carcinomas, especially in invasive areas (data not shown). Granular intracytoplasmic laminin immunoreactivity could be observed in some of the tumour cells in nine tumours, seven out of which represented grade III and two grade II transitional cell carcinomas (Figure 4b). No intracytoplasmic immunoreactivity for type IV collagen could be observed in any of the carcinomas.

The BMs of the blood vessels, smooth muscle cells and adipocytes stained positive for laminin and type IV collagen in the bladder wall. An apparent increase in the number of the blood vessels was seen beneath the dysplastic and neoplastic papillary epithelium (Figure 2b).

Seven of the p53 positive tumours showed areas of invasion. Thus, the degree of p53 positivity was related to the disruption of the BMs and the presence of invasion in the tumours. Six out of these seven tumours exhibited concomitant intracytoplasmic laminin immunoreactivity (Figures 4a and 4b).

### *Correlation of the p53 nuclear overexpression to the progression of bladder cancer*

We defined two groups of patients according to the different patterns of staining for p53 and compared the progression-free intervals in these groups using the Kaplan-Meier analysis (Figure 5). The rate of disease progression was higher in the group of patiens showing positive staining for p53 when compared to patients with negative p53 staining.

### Discussion

We found a 50% frequency of p53 positivity in our bladder carcinoma material. This concords with other investigations, where mutations of the p53 gene and immunohistochemical positivity for p53 protein have been found in 40–60% of transitional cell carcinomas (Olumi *et al.*, 1990; Sidransky *et al.*, 1991; Wright *et al.*, 1991). In this study all tumours associated with p53 positive dysplastic lesions were either of grade II or III (see Table III). Furthermore, p53 positivity in carcinoma material was clearly concentrated in tumours of higher grade and invasion (see Table I). Since generally in

**Figure 2** Immunoreactivity for p53 and type IV collagen in moderate dysplasia of the urinary bladder. **a**, The nuclei of the cells stain strongly for p53. **b**, A neighbouring section stained for type IV collagen. An intact BM is seen beneath the epithelium. Note also the increased vascularity beneath the epithelium, revealed by the type IV collagen stain (Immunoperoxidase stain, **a** & **b** × 260).

**Figure 3** p53 and type IV collagen immunoreactivity in a grade II transitional cell carcinoma. **a**, Strong staining for p53 is seen in the nuclei of the neoplastic cells. **b**, A type IV collagen positive BM can be seen beneath the papillary fronds of the tumour (Immunoperoxidase stain, **a** & **b** × 260).

**Figure 4** Immunoreactivity for p53 and laminin in a grade III transitional cell carcinoma **a**. p53 positive nuclei can be seen in the tumour cells. **b**, Intracytoplasmic laminin immunoreactivity is present in the cytoplasm of the tumour cells (Immunoperoxidase stain, **a** × 260, **b** × 510).

**Figure 5** Kaplan-Meier analysis of the progression-free survival in p53 positive and p53 negative cases showing a higher rate of urinary bladder carcinoma progression in patients expressing p53 positivity in the tumour compared to patients with negative p53 staining in the tumour.

normal cells p53 protein is undetectable by immunohistochemistry (Bartek *et al.*, 1990; Iggo *et al.*, 1990; Porter *et al.*, 1992; Barnes *et al.*, 1992), these results suggest that events leading to the accumulation of p53 protein play a part in the evolution of tumours of higher grade and occur in a preinvasive stage of the neoplastic epithelium.

According to the literature, positive p53 immunochemistry may be linked to several situations. It is well-documented that many mutations lead to an increased half-life of the p53 protein (Bartek *et al.*, 1990; Iggo *et al.*, 1990; Rodriques *et al.*, 1990). Thus, in many cases there has been a good correlation between mutational analysis and positive immunohistochemistry (Bartek *et al.*, 1991; Bennett *et al.*, 1992; Vähäkangas *et al.*, 1992). The half-life of p53 can also increase due to binding to some viral proteins as well as to the product of the mdm2 gene, which is often amplified in sarcomas (Vogelstein & Kinzler, 1992). Finally, mutated p53 may bind to wild type p53 and change it to the mutated conformation (Hainaut & Milner, 1992). Since conformation and oligomerisation of p53 is putatively important for the function, the function is probably changed in these cases as well (Vogelstein & Kinzler, 1992). This means that, whether due to mutation or other events, accumulation of the protein probably indicates a change of the state of the cell, as indicated by a non-mutated, but abundantly present p53 protein in a cancer family (Barnes *et al.*, 1992). Indeed, p53 immunohistochemistry has been suggested as an aid in diagnosis of malignancy (Hall *et al.*, 1991) and our current and earlier studies (Soini *et al.*, 1992) which show more positive cases among more aggressive tumours as well as other studies from the literature (Olumi *et al.*, 1990; Sidransky *et al.*, 1991) are in line with this suggestion.

Even though p53 immunoreactivity was mostly concentrated in grade II-III tumours there was, however, one p53 positive grade I tumour, and one p53 positive dysplastic lesion in which no associated carcinoma was found. If these cases harbour a mutated p53 protein it is possible that p53 mutations in this material are heterogenous in their nature and that corresponding proteins have behaved differentially. It has been shown that different mutant alleles have distinct biological properties in experimental systems (Levine, 1992). On the other hand, a p53 event may be an early change in tumours and take part in the transformation of the tumour to a more malignant type. However, the only dysplastic lesion preceding carcinoma (case 3) was p53 positive but the tumour was negative. The low percentage of positive cells in this case (up to 5%) may indicate a wild type rather than mutated p53 (Lu et al., 1992).

Administration of N-butyl-N-(4-hydroxybutyl)nitrosamine to mice causes changes from dysplasia to invasive carcinoma in a dose-dependent way suggesting that evolution of carcinoma in the bladder is a sequential process through these stages (Ohtani et al., 1986). However, dysplasia of the bladder epithelium in man is often detected in association with rather than preceeding a carcinoma (Murphy, 1989). As in our study, such dysplasia is frequently associated with invasive and aggressive types of transitional cell carcinoma, and dysplastic lesion in bladders treated for carcinoma may predict a recurrence of the disease (Murphy, 1989; Wolf et al., 1985; Kakizoe et al., 1985). The close association reported here between expression of p53 protein in dysplasia and the related transitional cell carcinomas suggests that these processes may be linked together in mechanism. In an analogous situation in the bronchus, preinvasive and microinvasive lesions adjacent to an invasive squamous cell carcinoma all contained the same p53 mutations (Vähäkangas et al., 1992). Whether individual cases share similar mutations of the p53 gene in bladder carcinomas and dysplastic lesions remains to be determined.

Our findings of the immunohistochemical distribution of BMs and intracytoplastic laminin immunoreactivity in transitional cell carcinomas are in accordance with the general observation in other types of tumours (Martinez-Hernandez & Amenta, 1983; Bosman et al., 1985); the more malignant and aggressive the tumour is, the more usual is also the disruption of BMs around the tumour islands and intracytoplasmic laminin immunoreactivity of tumour cells. It has also been shown that BM disruption in bladder carcinomas correlates with lower 5-year survival rate, higher tumour stage, higher histological grade and tumour ploidy (Schapers et al., 1990). In this material BM disruption was also associated with positive p53 immunohistochemistry, further emphasising the fact that p53 positive bladder carcinomas are of a more aggressive nature. The increased intracytoplasmic laminin immunoreactivity in high grade tumours reflects the increased synthesis of BM proteins due to the disruption of the BMs.

Carcinogenesis is a multistage process in which accumulation of chromosomal changes eventually leads to a development of a malignant tumour (Fearon & Vogelstein, 1990). In addition to p53 gene changes, several other genetic changes, such as deletion of chromosomes 9 and 11 (Olumi et al., 1990; Sidransky et al., 1991), activation of ras and c-*erb*B-2 (Santos et al., 1982; Wright et al., 1991) and inactivation of the retinoblastoma gene (Ishikawa et al., 1991), have been observed in bladder carcinomas. Thus a p53 mutation, even though present in a large number of transitional cell carcinomas, represents only one event in the putative pathway of evolution of these tumours. Because p53 mutations are not found in all tumours (Sidransky et al., 1991), other pathways must exist, not requiring p53 mutations at all.

In conclusion, our results show that p53 protein expression can frequently be found in dysplastic lesions following the treatment of bladder carcinoma. Since they are associated with aggressive and recurrent tumours which also harbour a high rate of p53 protein expression, p53 gene mutations possibly play a role in the evolution of tumours of a higher grade. It may be possible to use p53 protein immunohistochemistry as an adjunct in the assessment and follow-up of epithelial changes in patients with urothelial carcinoma.

The p53 antibody CM-1 was kindly provided by Dr David Lane (Cancer Research Campaign Laboratory, Medical Sciences Institute, University of Dundee, UK). This study was supported financially by The Finnish Cultural Fund, The Finnish Anti-Tuberculosis Association, The Finnish Cancer Society, The Paulo Foundation and the CIMO (Centre for International Mobility) of Finland.

### References

BARNES, D.M., HANBY, A.M., GILLETT, C.E., MOHAMMED, S., HODGSON, S., BORROW, L.G., LEIGH, I.M., PURKIS, T., MACGEOCH, C., SPURR, N.K., BARTEK, J., VOGTESEK, B., PICKSLEY, S.M. & LANE, D.P. (1992). Abnormal expression of wild type p53 protein in normal cells of a cancer family patient. *Lancet*, **340**, 259-263.

BARTEK, J., BARTKOVA, J., VOJTESEK, B., STASKOVA, Z., LUKAS, J., REJTHAR, A., KOVARIK, J., MIDGLEY, C.A., GANNON, J.V. & LANE, D.P. (1991). Aberrant expression of the p53 oncoprotein is a common feature of a wide spectrum of human malignancies. *Oncogene*, **6**, 1699-1703.

BARTKOVA, J., BARTEK, J., LUKAS, J., VOITESEK, B., STASKOVA, Z., REJTHAR, A., KOVARIK, J., MIDGLEY, C.A. & LANE, D.P. (1991). p53 protein alterations in human testicular cancer including pre-invasive intratubular germ-cell neoplasia. *Int. J. Cancer*, **49**, 196-202.

BENNETT, W.P., HOLLSTEIN, M.C., HE, A., ZSU, S.M., RESAU, J.H., TRUMP, B.F., METCALF, R.A., WELSH, J.A., MIDGLEY, C., LANE, D.P. & HARRIS, C.C. (1991). Archival analysis of p53 genetic and protein alterations in Chinese esophageal cancer. *Oncogene*, **6**, 1779-1784.

BISCHOFF, J.R., FRIEDMAN, P.N., MARSHAK, D.R., PRIVES, C. & BEACH, D. (1990). Human p53 is phosphorylated by p64-cdc2 and cyclin B-cdc2. *Proc. Natl Acad. Sci. USA*, **87**, 4766-4770.

BOSMAN, F.T., HAVENITH, M. & CLEUTJENS, J.P.M. (1985). Basement membranes in cancer. *Ultrastruct. Pathol.*, **8**, 291-304.

CASSON, A.G., MUKHOPADHYAY, T., CLEARY, K.R., RO, J.Y., LEVIN, B. & ROTH, J.A. (1991). p53 gene mutations in Barrett's epithelium and esophageal cancer. *Cancer Res.*, **51**, 4495-4499.

CHIBA, I., TAKAHASHI, T., NAU, M.M., D'AMICO, D., CURIEL, D.T., MITSUDOMI, T., BUCHHAGEN, D.L., CARBONE, D., PIANTADOSI, S., KOGA, H., REISSMAN, P.T., SLAMON, D.J., HOLMES, E.C. & MINNA, J.D. (1990). Mutations in the p53 gene are frequent in primary, resected non-small cell lung cancer. *Oncogene*, **5**, 1603-1610.

DEPPERT, W., BUSCHHAUSEN-DENKER, G., PATSCHINSKY, T. & STEINMEYER, K. (1990). Cell cycle control of p53 in normal (3T3) and chemically transformed (Meth A) mouse cells. II. Requirement for cell cycle progression. *Oncogene*, **5**, 1701-1706.

EKBLOM, P., MIETTINEN, M., RAPOLA, J. & FOIDART, J.M. (1982). Demonstration of laminin, a basement membrane glycoprotein in routine processed formalin fixed human tissues. *Histochemistry*, **75**, 301-309.

ELIYAHU, D., MICHALOVITZ, D., ELIYAHU, S., PINHASI-KIMHI, O. & OREN, M. (1989). Wild-type p53 can inhibit oncogene-mediated focus formation. *Proc. Natl Acad. Sci. USA*, **86**, 8763-8767.

FARMER, G., BARGONETTI, J., ZHU, H., FRIEDMAN, P., PRYWES, R. & PRIVES, C. (1992). Wild-type p53 activates transcription *in vitro*. *Nature*, **358**, 83-86.

FEARON, E.R. & VOGELSTEIN, B. (1990). A genetic model for colorectal tumorigenesis. *Cell*, **61**, 759-767.

FINLAY, C.A., HINDS, P.W. & LEVINE, A.J. (1989). The p53 proto-oncogene can act as a suppressor of transformation. *Cell*, **57**, 1083-1093.

GUSTERSON, B.A., ANBAZHAGEN, R., WARREN, W., MIDGLEY, C., LANE, D.P., O'HARE, M., STAMPS, A., CARTER, R. & JAYATILAKE, H. (1991). Expression of p53 in premalignant and malignant squamous epithelium. *Oncogene*, **6**, 1785-1789.

HAINAUT, P. & MILNER, J. (1992). Interaction of heat-shock protein 70 with p53 translated *in vitro*: evidence for interaction with dimeric p53 and for the role in the regulation of p53 conformation. *EMBO J.*, **11**, 3513-3520.

HALEVY, O., RODEL, J., PELED, A. & OREN, M. (1991). Frequent p53 mutations in chemically induced murine fibrosarcoma. *Oncogene*, **6**, 1593-1600.

HALL, P.A., RAY, A., LEMOINE, N.R., MIDGLEY, C.A., KRAUSZ, T. & LANE, D.P. (1991). p53 immunostaining as a marker of malignant disease in diagnostic cytopathology. *Lancet*, **338**, 513.

HOLLSTEIN, M., SIDRANSKY, D., VOGELSTEIN, B. & HARRIS, C. (1991). p53 mutations in human cancers. *Science*, **253**, 49-53.

HSU, S.M., RAINE, L. & FANGER, H. (1981). Use of avidin-biotin-peroxidase complex (ABC) in immunoperoxidase techniques: a comparison between ABC and unlabelled antibody (PAP) procedures. *J. Histochem. Cytochem.*, **29**, 577-580.

IGGO, R., GATTER, K., BARTEK, J., LANE, D. & HARRIS, A.L. (1990). Increased expression of mutant forms of p53 oncogene in primary lung cancer. *Lancet*, **335**, 675-679.

ISHIKAWA, J., XU, H.-J., YANDELL, D.W., MAEDA, S., KAMIDONO, S., BENEDICT, W.F. & TAKAHASHI, R. (1991). Inactivation of the retinoblastoma gene in human bladder and renal cell carcinomas. *Cancer Res.*, **51**, 5736-5743.

KAKIZOE, T., MATUMOTO, K., NISHIO, Y., OHTANI, M. & KISHI, K. (1985). Significance of carcinoma *in situ* and dysplasia in association with bladder cancer. *J. Urol.*, **133**, 395-398.

LANE, D. & BENCHIMOL, S. (1990). p53: oncogene or anti-oncogene. *Genes Dev.*, **4**, 1-8.

LEVINE, A.J. (1992). The p53 tumour suppressor gene and product. *Cancer Surveys. Volume 12: Tumour Suppressor Genes, the Cell Cycle and Cancer*. pp. 59-79.

LU, X., PARK, S.H., THOMPSON, T.C. & LANE, D.P. (1992). ras-induced hyperplasia occurs with mutation of p53, but activated ras and myc together can induce carcinoma without p53 mutation. *Cell*, **70**, 153-161.

MARTINEZ-HERNANDEZ, A. & AMENTA, P.S. (1983). The basement membrane in pathology. *Lab. Invest.*, **48**, 656-677.

MAZARS, R., PUJOL, P., MAUDELONDE, T., JEANTEUR, P. & THEILLET, C. (1991). p53 mutations in ovarian cancer: a late event? *Oncogene*, **6**, 1685-1690.

MERCER, W.E., AVIGNOLO, C. & BASEGRA, R. (1984). Role of p53 protein in cell proliferation as studied by microinjection of monoclonal antibodies. *Mol. Cell. Biol.*, **4**, 276-281.

MIDGLEY, C.A., FISHER, C.J., BARTEK, J., VOJTESEK, B., LANE, D. & BARNES, D.M. (1992). Expression of human p53 in bacteria: application to the analysis of p53 expression in human tumors. *J. Cell. Sci.*, **101**, 183-189.

MILNER, J. (1991). The of p53 in normal control of cell proliferation. *Curr. Opin. Cell. Biol.*, **3**, 282-286.

MOSTOFI, F.K., SOBIN, L.H. & TORLONI, H. (1973). Histologic typing of urinary bladder tumors. In *International Histological Classification of Tumours*. No. 10. World Health Organization: Geneva.

MURPHY, W.M. (1989). *Urological Pathology*. W.B. Saunders Company: Philadelphia, pp. 34-146.

NAGY, G.K., FRABLE, W.J. & MURPHY, W.M. (1982). Classification of premalignant urothelial abnormalities. A Delphi study of the National Bladder Cancer Collaborative Group A. *Path. Ann.*, **17**, 219-233.

NIGRO, J.M., BAKER, S.J., PREISINGER, A.C., JESSUP, J.M., HOSTETTER, R., CLEARLY, K., BIGNER, S.H., DAVIDSON, N., BAYLIN, S., DEVILEE, P., GLOVER, T., COLLINS, F.S., WESTON, A., MODALI, R., HARRIS, C.C. & VOGELSTEIN, B. (1989). Mutations in the p53 gene occur in diverse human tumour types. *Nature*, **342**, 705-708.

NUORVA, K., SOINI, Y., KAMEL, D., AUTIO-HARMAINEN, H., RISTELI, L., RISTELI, J., VÄHÄKANGAS, K. & PÄÄKKÖ, P. (1993). Concurrent p53 expression in bronchial dysplasias and squamous cell lung carcinomas. *Am. J. Pathol.*, **142**, 725-732.

OHTANI, M., KAKIZOE, T., NISHIO, Y., SATO, S., SUGIMURA, T., FUKUSHIMA, S. & NIIJIMA, T. (1986). Sequential changes of mouse bladder epithelium during induction of invasive carcinomas by N-butyl-N-(4-hydroxybutyl)nitrosamine. *Cancer Res.*, **46**, 2001-2004.

OLUMI, A.F., TSAI, Y.C., NICHOLS, P.W., SKINNER, D.G., CAIN, D.R., BENDER, L.I. & JONES, P.A. (1990). Allelic loss of chromosome 17p distinguishes high grade from low grade transitional cell carcinomas of the bladder. *Cancer Res.*, **50**, 7081-7083.

PORTER, P.L., GOWN, A.M., KRAMP, S.G. & COLTRERA, M.D. (1992). Widespread p53 overexpression in human malignant tumors. An immunohistochemical study using methacarn-fixed, embedded tissue. *Am. J. Pathol.*, **140**, 145-153.

PURDIE, C.A., O'GRADY, J., PIRIS, J., WYLLIE, A.H. & BIRD, C.C. (1991). p53 expression in colorectal tumors. *Am. J. Pathol.*, **138**, 807-813.

RISINGER, J., DENT, G., IGNAR-TROWBRIDGE, D., MCLACHLAN, J., TSAO, M.-S., SENTERMAN, M. & BOYD, J. (1992). p53 gene mutations in human endometrial carcinoma. *Molecular Carcinogenesis*, **5**, 250-253.

RISTELI, J., BÄCHINGER, H.P., ENGEL, J., FURTHMAYR, H. & TIMPL, R. (1980). 7-S collagen: characterization of an unusual basement membrane structure. *Eur. J. Biochem.*, **108**, 239-250.

RISTELI, J. & TIMPL, R. (1981). Isolation and characterization of pepsin fragments of laminin from human placenta and renal basement membranes. *Biochem. J.*, **193**, 749-755.

RODRIGUES, N., ROWAN, A., SMITH, M.E., KERB, I.B., BODMER, W.F., GANNON, J.V. & LANE, D.P. (1990). p53 mutations in colorectal cancer. *Proc. Natl Acad. Sci. USA*, **87**, 7555-7559.

SANTOS, E., TRONICK, S.R., AARONSON, S.A., PULCIANI, S. & BARBACID, M. (1982). T24 human bladder carcinoma oncogene is an activated form of the normal human homologue of BALB- and Harvey-MSV transforming genes. *Nature*, **298**, 343-347.

SCHAPERS, R.F., PAUWELS, R.P., HAVENITH, M.G., SMEETS, A.W., VAN DEN BRANDT, P.A. & BOSMAN, F.T. (1990). Prognostic significance of type IV collagen and laminin immunoreactivity in urothelial carcinomas of the bladder. *Cancer*, **66**, 2583-2588.

SIDRANSKY, D., VON ESCHENBACH, A., TSAI, Y.C., JONES, P., SUMMERHAYES, I., MARSHALL, F., PAUL, M., GREEN, P., HAMILTON, S.R., FROST, P. & VOGELSTEIN, B. (1991). Identification of p53 gene mutations in bladder cancers and urine samples. *Science*, **25**, 705-709.

SIMON, R.M. (1989). Design and conduct of clinical trials. In DeVita Jr, V.T., Hellman, S. & Rosenberg, S.A. *Cancer. Principles and Practice of Oncology*. Vol. 1. Lippincott, Philadelphia, Third Edition, pp. 396-420.

SOINI, Y., PÄÄKKÖ, P., NUORVA, K., KAMEL, D., LANE, D.P. & VÄHÄKANGAS, K. (1992). Comparative analysis of p53 protein immunoreactivity in prostatic, lung and breast carcinomas. *Virchows Arch. A.*, **421**, 223-228.

STEINMEYER, K., MAACKE, H. & DEPPERT, W. (1990). Cell cycle control by p53 in normal (3T3) and chemically transformed (Meth A) mouse cells. I. Regulation of p53 expression. *Oncogene*, **5**, 1691-1699.

VOGELSTEIN, B. (1989). Chromosome 17 deletions and p53 gene mutations in colorectal carcinomas. *Science*, **244**, 217-221.

VOGELSTEIN, B. & KINZLER, K.W. (1992). p53 function and dysfunction. *Cell*, **70**, 523-526.

WOLF, H., OLSEN, P.R. & HOJGAARD, K. (1985). Urothelial dysplasia concomitant with bladder tumours: a determinant for future new occurrences in patients treated by full-course radiotherapy. *Lancet*, **1**, 1005-1008.

WRIGHT, C., MELLON, K., JOHNSTON, P., LANE, D.P., HARRIS, A.L., HORNE, C.H.W. & NEAL, D.E. (1991). Expression of mutant p53, c-*erb*B-2 and the epidermal growth factor receptor in transitional cell carcinoma of the human urinary bladder. *Br. J. Cancer*, **63**, 967-970.

WRIGHT, P.A., LEMOINE, N.R., GORETZKI, P.E., WYLLIE, F.S., BOND, J., HUGHES, C., RÖHER, H.-D., WILLIAMS, E.D. & WYNFORD-THOMAS, D. (1991). Mutations of the p53 gene in a differentiated human thyroid carcinoma cell line, but not in primary thyroid tumors. *Oncogene*, **6**, 1693-1697.

VÄHÄKANGAS, K.H., SAMET, J.M., METCALF, R.A., WELSH, J.A., BENNETT, W.P., LANE, D.P. & HARRIS, C.C. (1992). Mutations of p53 and ras genes in radon-associated lung cancer from uranium miners. *Lancet*, **339**, 576-580.